# Supplementary material for: N-Acetylation of Amines in Continuous-Flow with Acetonitrile—No Need for Hazardous and Toxic Carboxylic Acid Derivatives
Source: Molecules. 2020 Apr 23;25(8):1985. doi: 10.3390/molecules25081985 (PMC7221708; doi:10.3390/molecules25081985)
Supplement: Supplementary file 1 [file molecules-25-01985-s001.pdf]

molecules

N-Acetylation of Amines in Continuous-Flow with Acetonitrile–No Need for Hazardous and Toxic Carboxylic Acid Derivatives

György Orsy [1,2], Ferenc Fülöp [1,3,*] and István M. Mándity [2,4,*]

1 Institute of Pharmaceutical Chemistry University of Szeged, Eötvös u. 6, H-6720 Szeged, Hungary; orsy.gyorgy@ttk.hu (G.O.)
2 MTA TTK Lendület Artificial Transporter Research Group, Institute of Materials and Environmental Chemistry, Research Center for Natural Sciences, Hungarian Academy of Sciences, Magyar Tudosok krt. 2, 1117 Budapest, Hungary
3 Research Group of Stereochemistry of the Hungarian Academy of Sciences, Dóm tér 8, H-6720 Szeged, Hungary
4 Department of Organic Chemistry, Faculty of Pharmacy, Semmelweis University, Hőgyes Endre u. 7, H-1092, Budapest, Hungary
* Correspondence: fulop@pharm.u-szeged.hu (F.F.); mandity.istvan@ttk.mta.hu (I.M.M.); Tel.: +36 1 3826 616 (I.M.M.)

Table of Content

1. 1H and 13C NMR spectra S3 – S13

2. Images of the home made reactor S14

1. NMR spectra

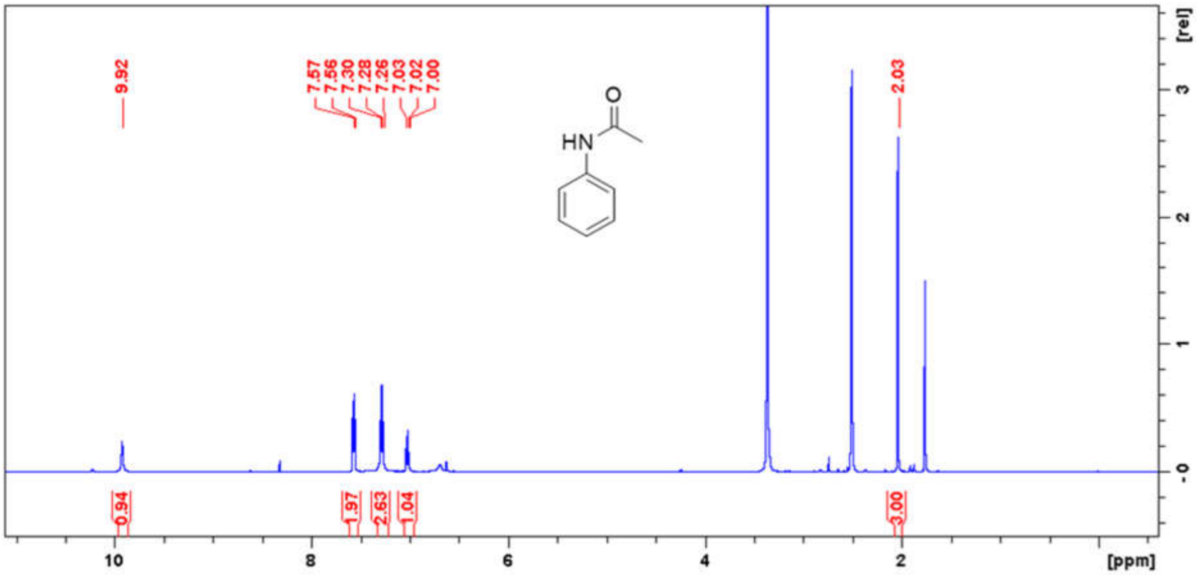

Figure S1. ^{1}H NMR spectrum of acetanilide **2** measured in DMSO-d_6 at 298 K.

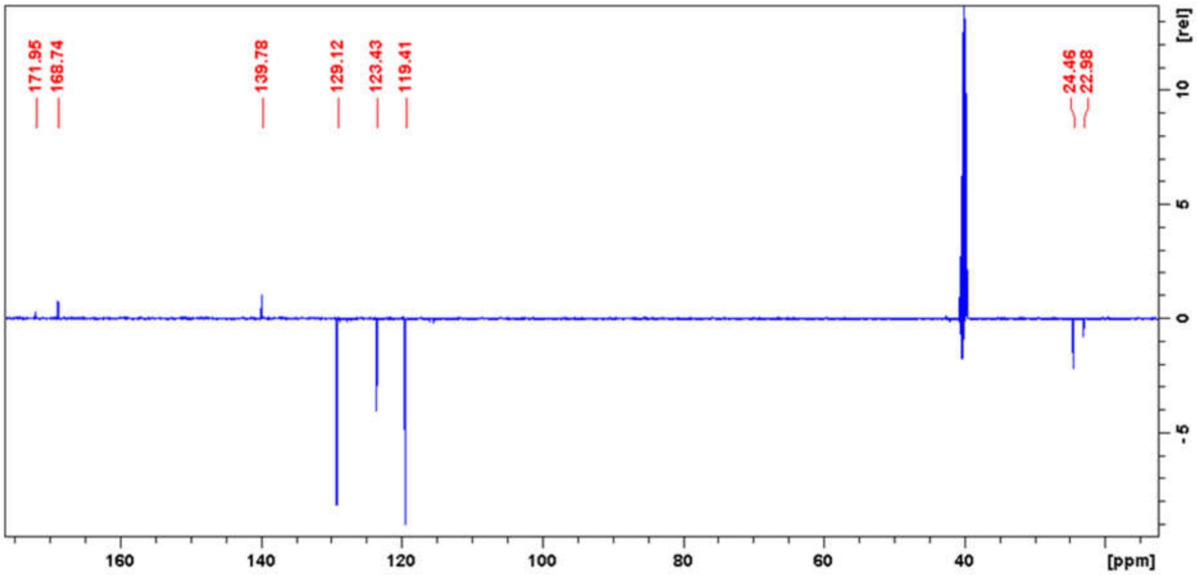

Figure S2. APT NMR spectrum of acetanilide **2** measured in DMSO-d_6 at 298 K.

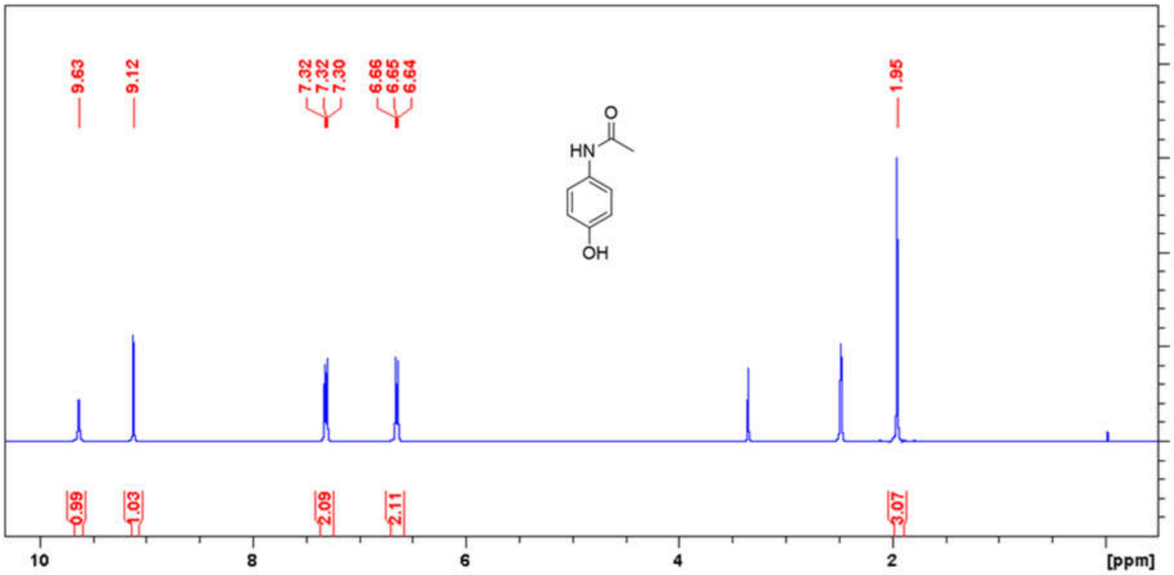

Figure S3. ^{1}H NMR spectrum of acetaminophen **4** measured in DMSO-d_6 at 298 K.

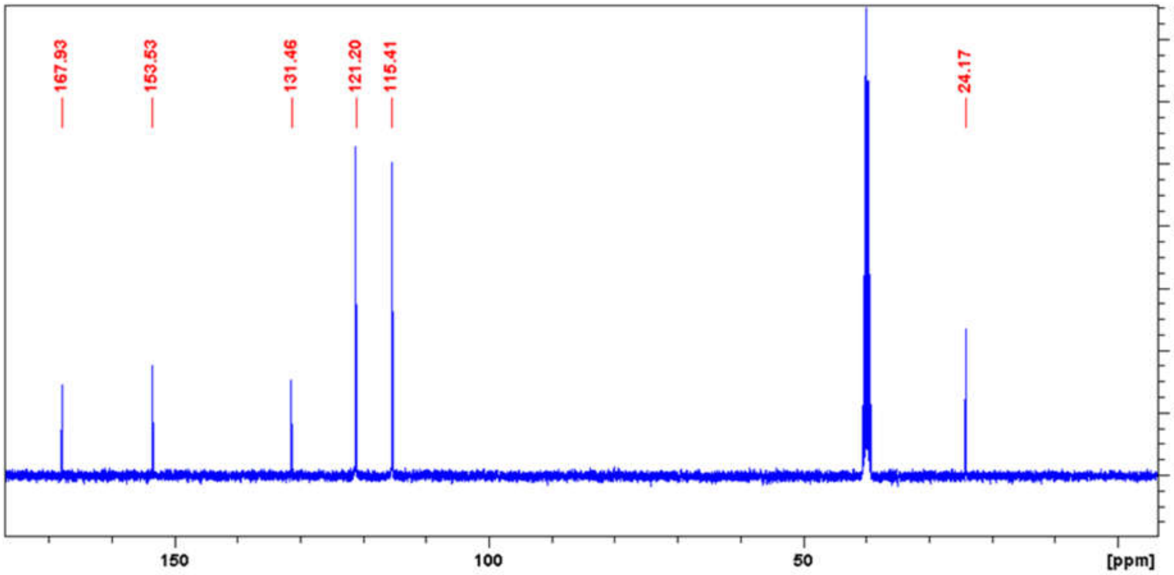

Figure S4. ^{13}C NMR spectrum of acetaminophen **4** measured in DMSO-d_6 at 298 K.

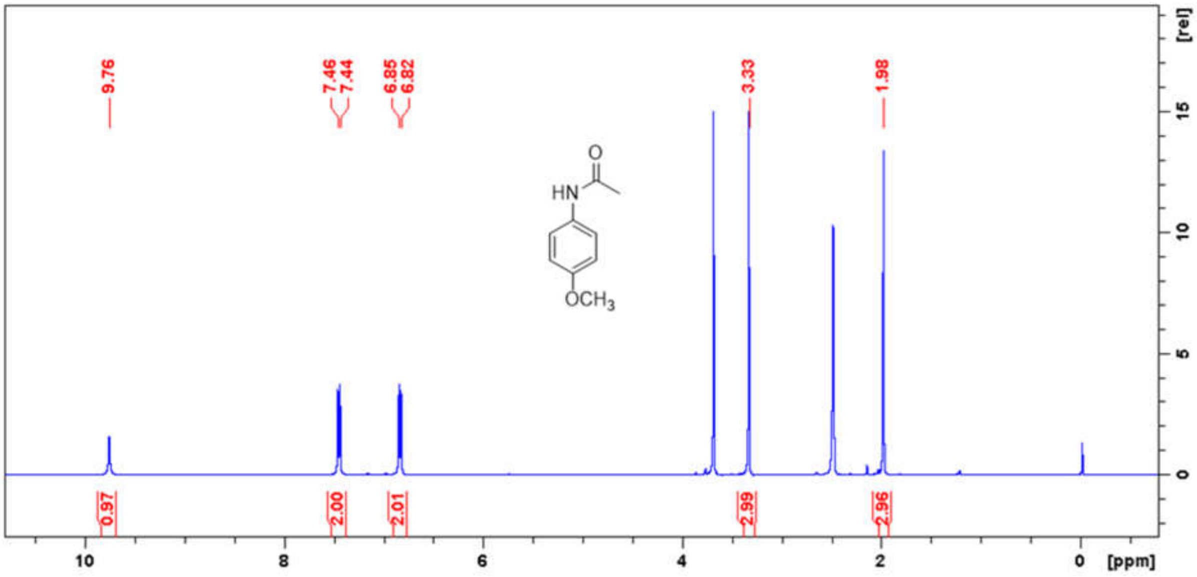

Figure S5. ^{1}H NMR spectrum of N-(4-methoxyphenyl)acetamide **6** measured in DMSO-d_6 at 298 K.

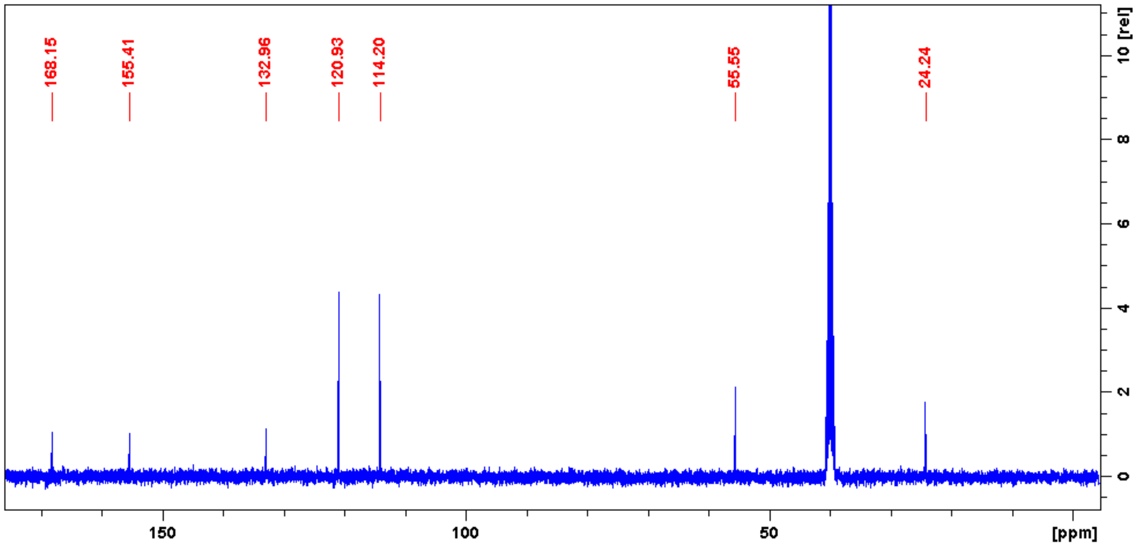

Figure S6. ^{13}C NMR spectrum of N-(4-methoxyphenyl)acetamide **6** measured in DMSO-d_6 at 298 K.

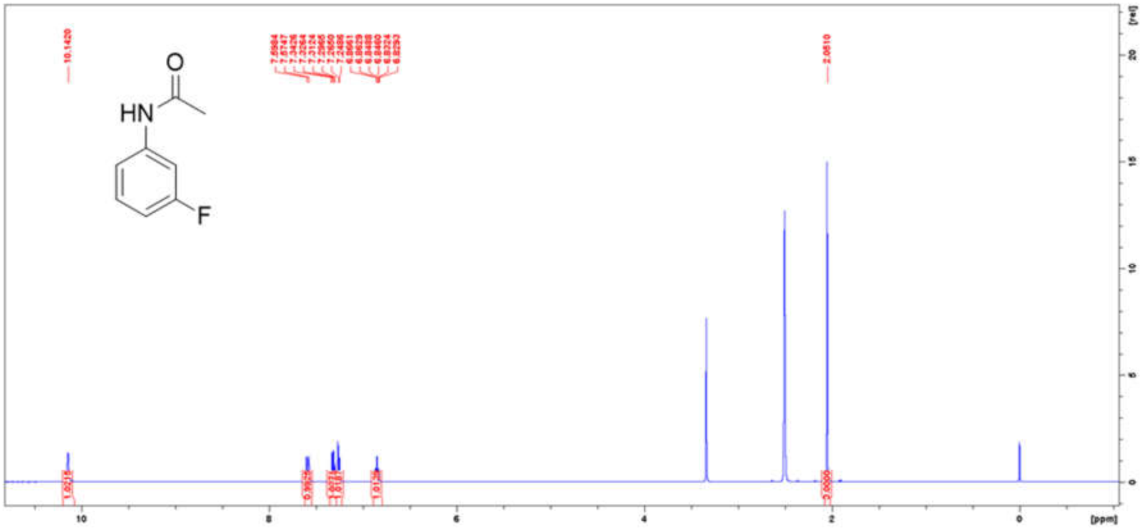

Figure S7. ^{1}H NMR spectrum of N-(3-fluorophenyl)acetamide **8** measured in DMSO-d_6 at 298 K.

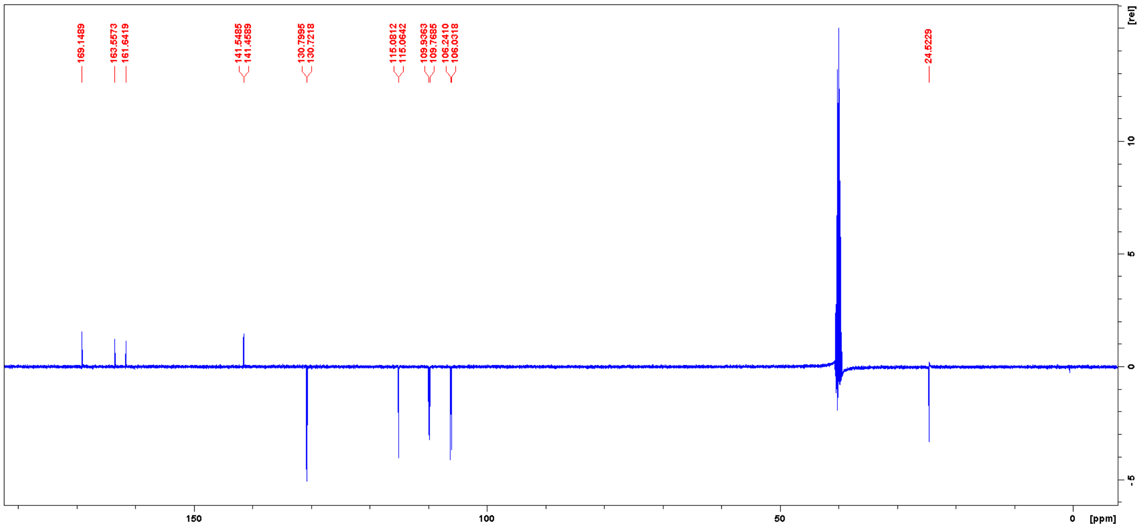

Figure S8. ^{13}C NMR spectrum of N-(3-fluorophenyl)acetamide **8** measured in DMSO-d_6 at 298 K.

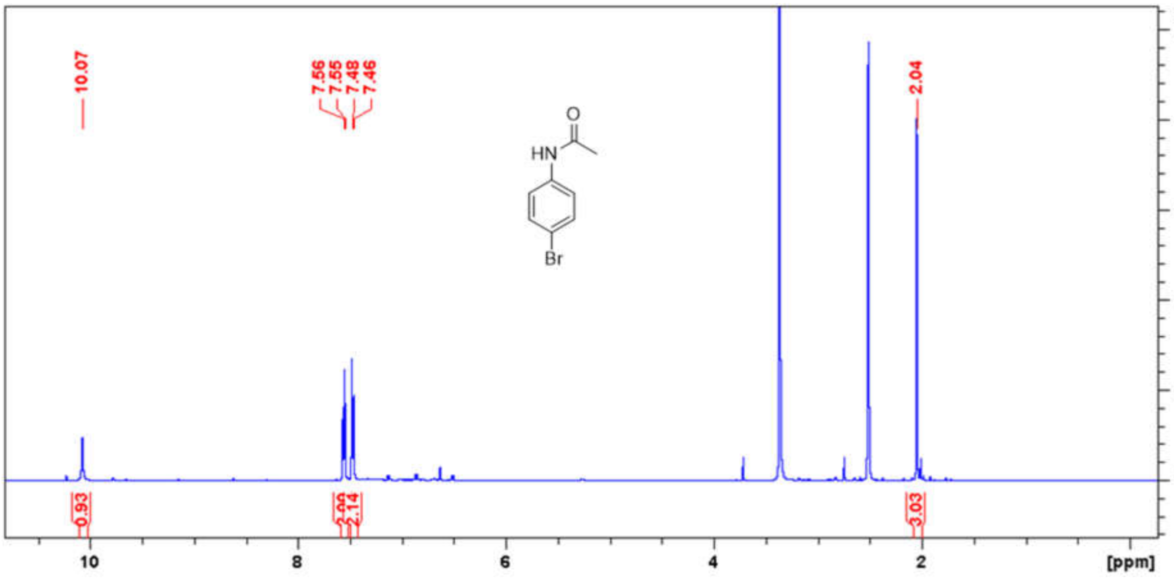

Figure S9. ^{1}H NMR spectrum of N-(4-Bromophenyl)acetamide **10** measured in DMSO-d_6 at 298 K.

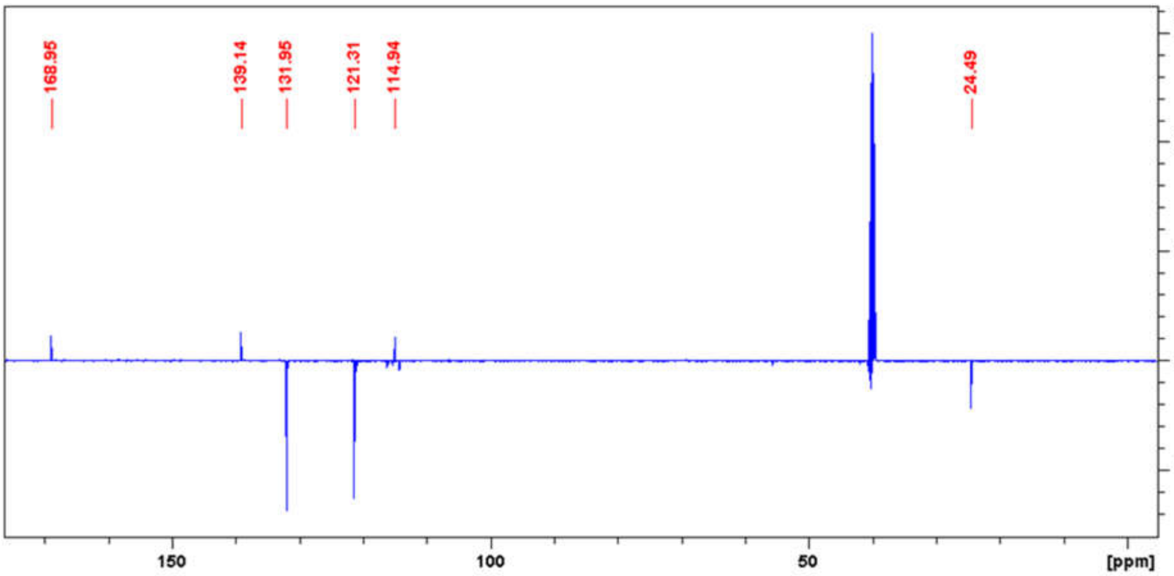

Figure S10. APT NMR spectrum of N-(4-Bromophenyl)acetamide **10** measured in DMSO-d_6 at 298 K.

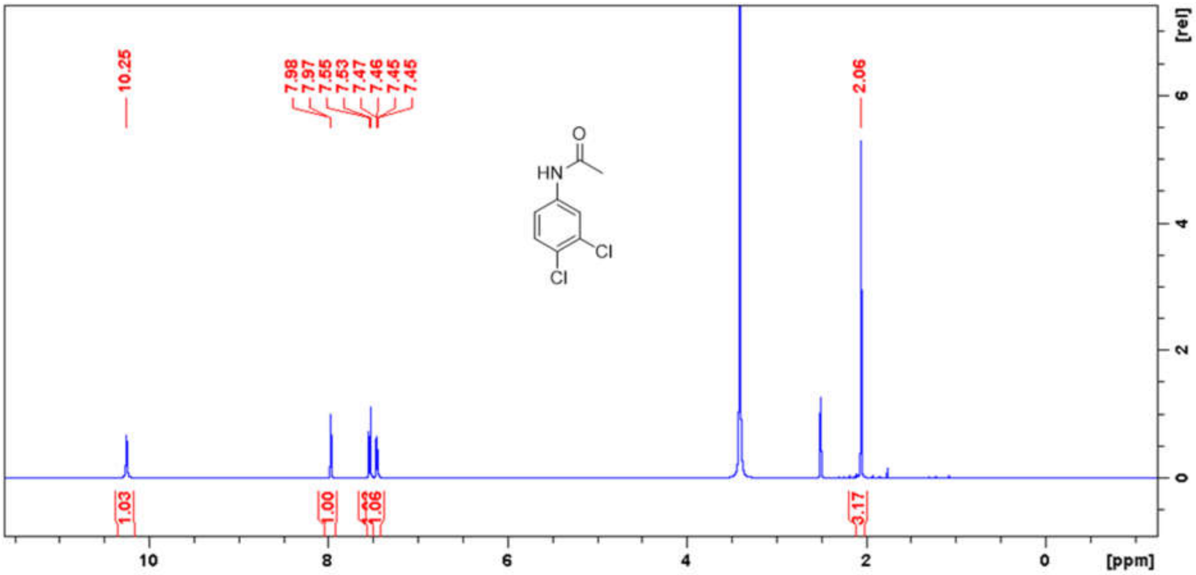

Figure S11. ^{1}H NMR spectrum of N-(3,4-dichlorophenyl)acetamide **12** measured in DMSO-d_6 at 298 K.

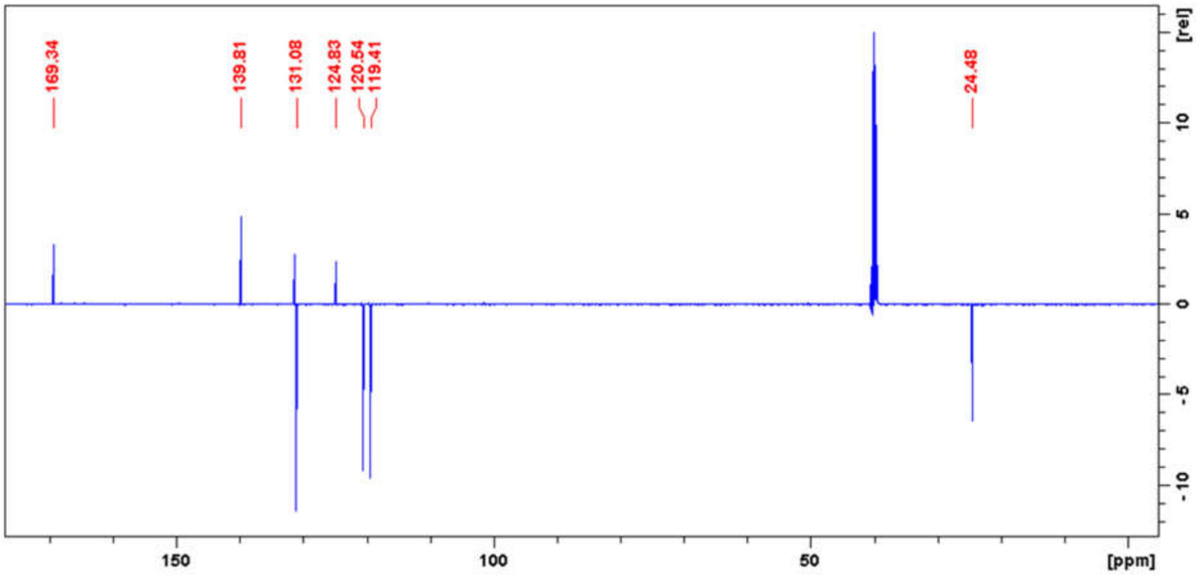

Figure S12. APT NMR spectrum of N-(3,4-dichlorophenyl)acetamide **12** measured in DMSO-d_6 at 298K.

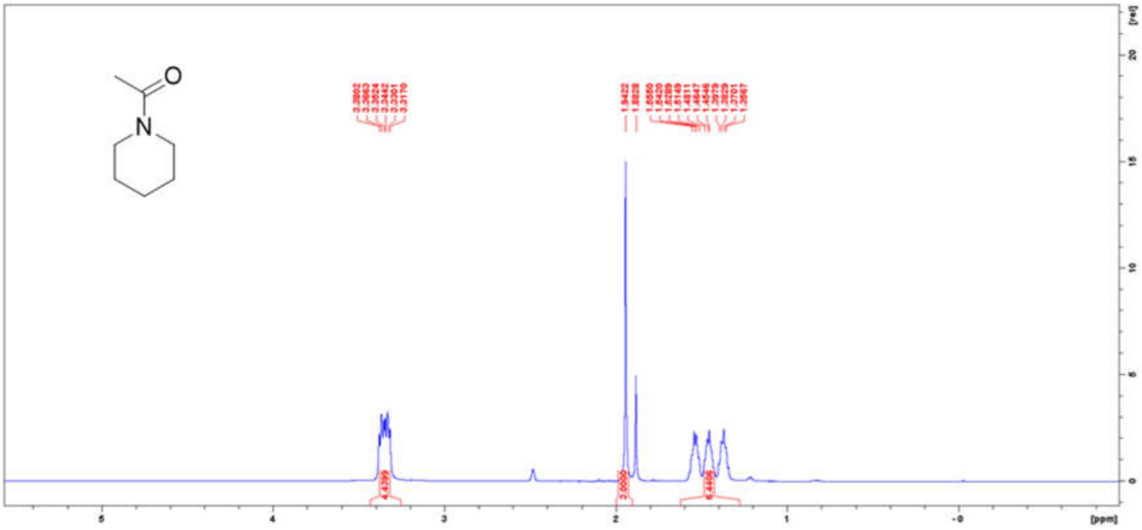

Figure S13. ^{1}H NMR spectrum of 1-acetylpiperidine **20** measured in DMSO-d_6 at 298 K.

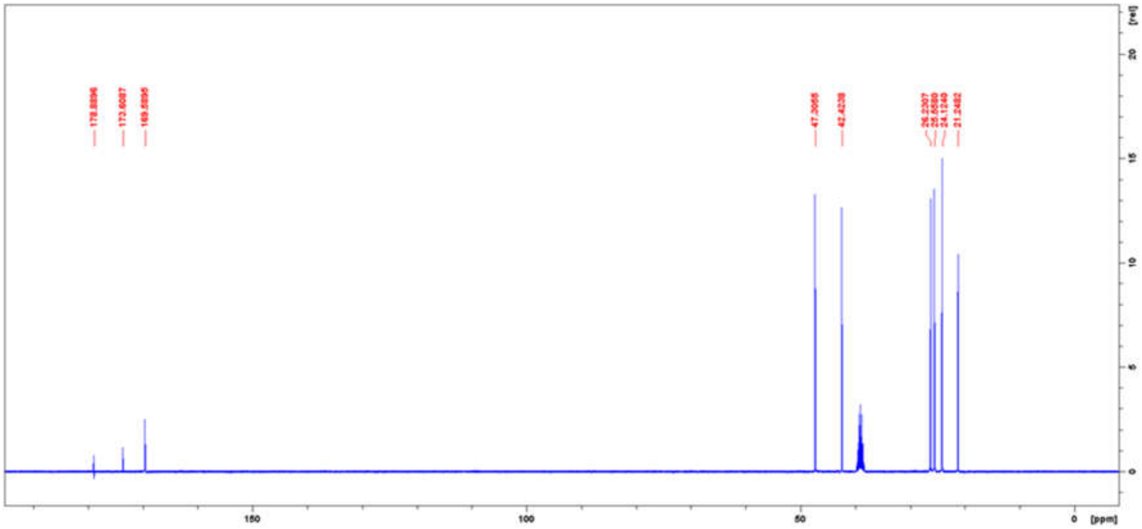

Figure S14. ^{13}C NMR spectrum of 1-acetylpiperidine **20** measured in DMSO-d_6 at 298 K.

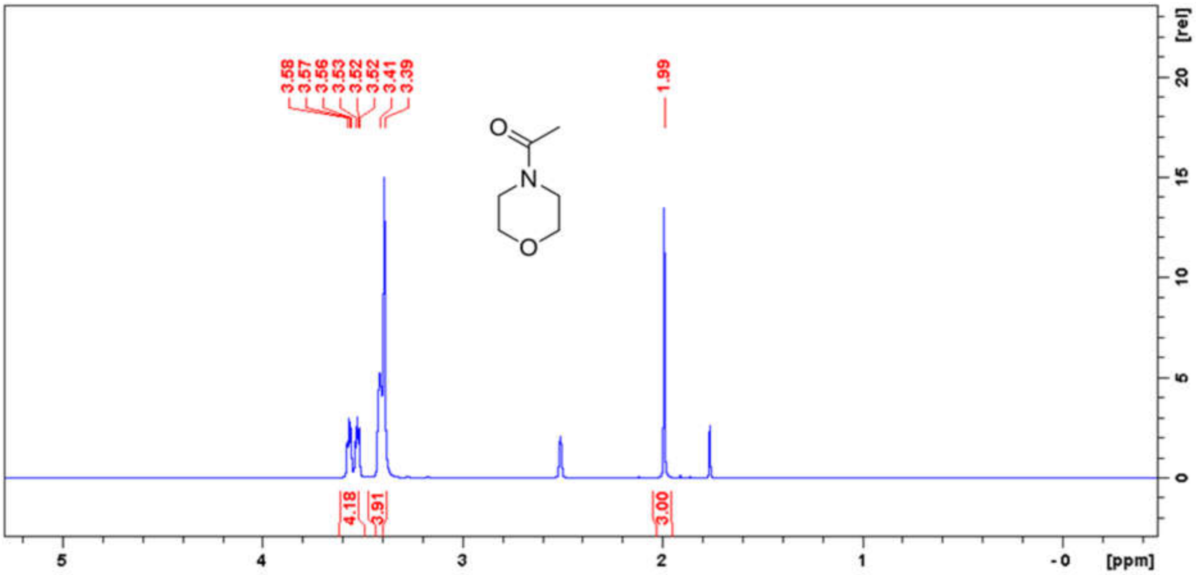

Figure S15. ^{1}H NMR spectrum of 4-acetylmorpholine **22** measured in DMSO-d_6 at 298 K.

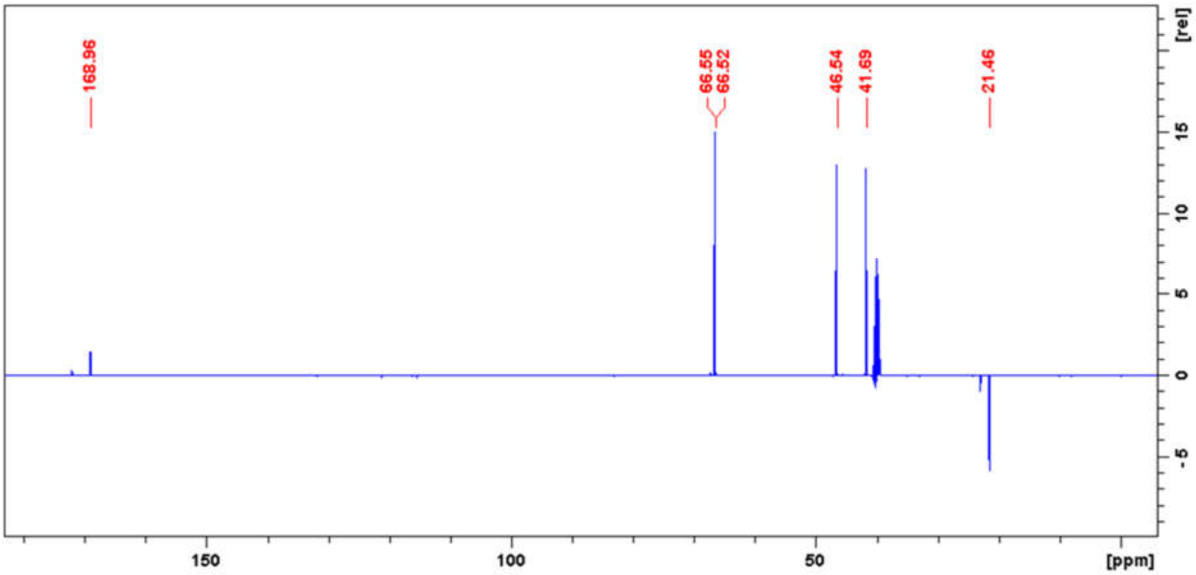

Figure S16. APT NMR spectrum of 4-acetylmorpholine **22** measured in DMSO-d_6 at 298 K.

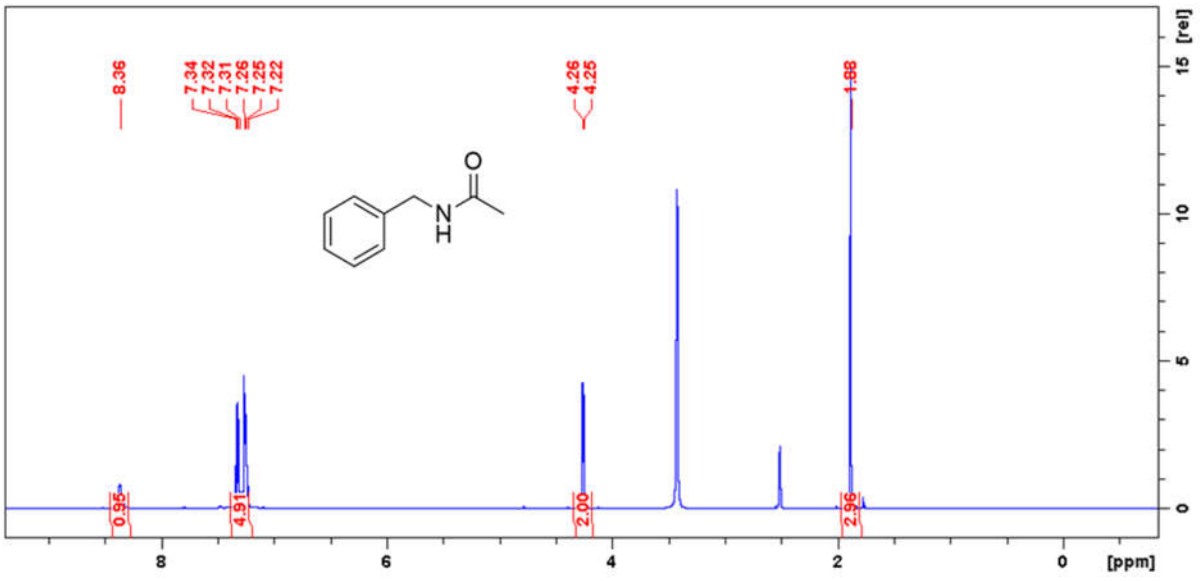

Figure S17. ^{1}H NMR spectrum of N-benzylacetamide **24** measured in DMSO-d_6 at 298 K.

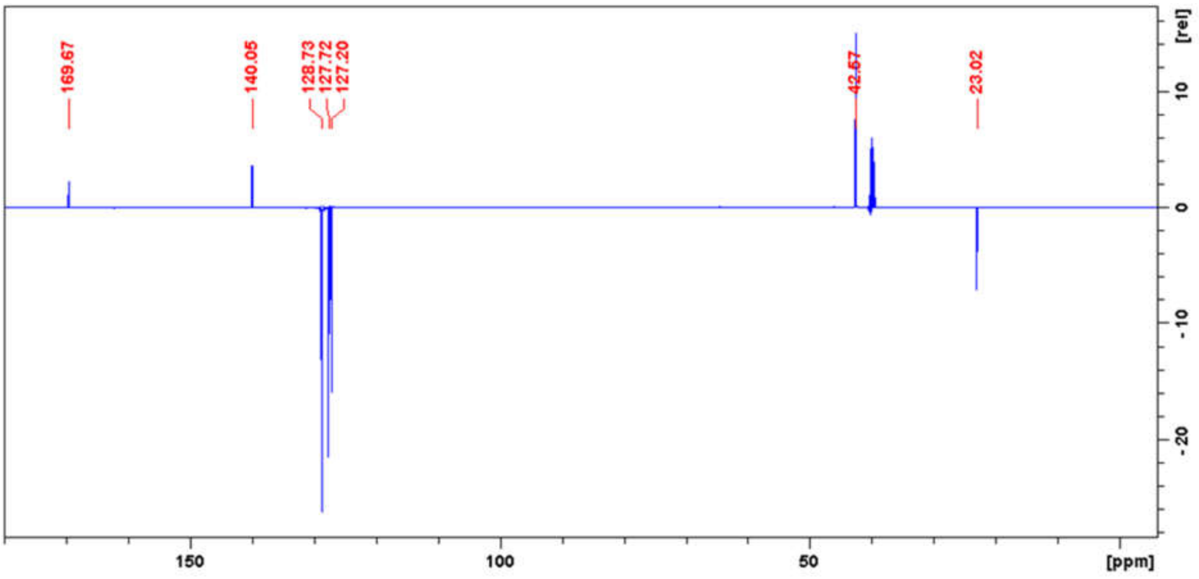

Figure S18. APT NMR spectrum of N-benzylacetamide **24** measured in DMSO-d_6 at 298 K.

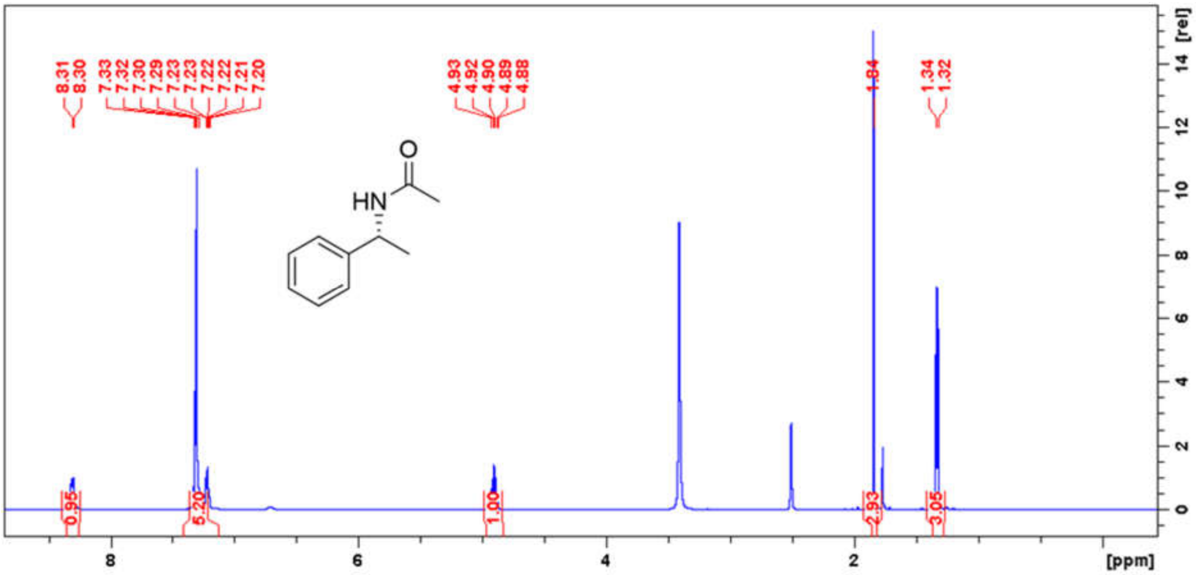

Figure S19. ^{1}H NMR spectrum of (R)-N-(1-phenylethyl)acetamide **26** measured in DMSO-d_6 at 298 K.

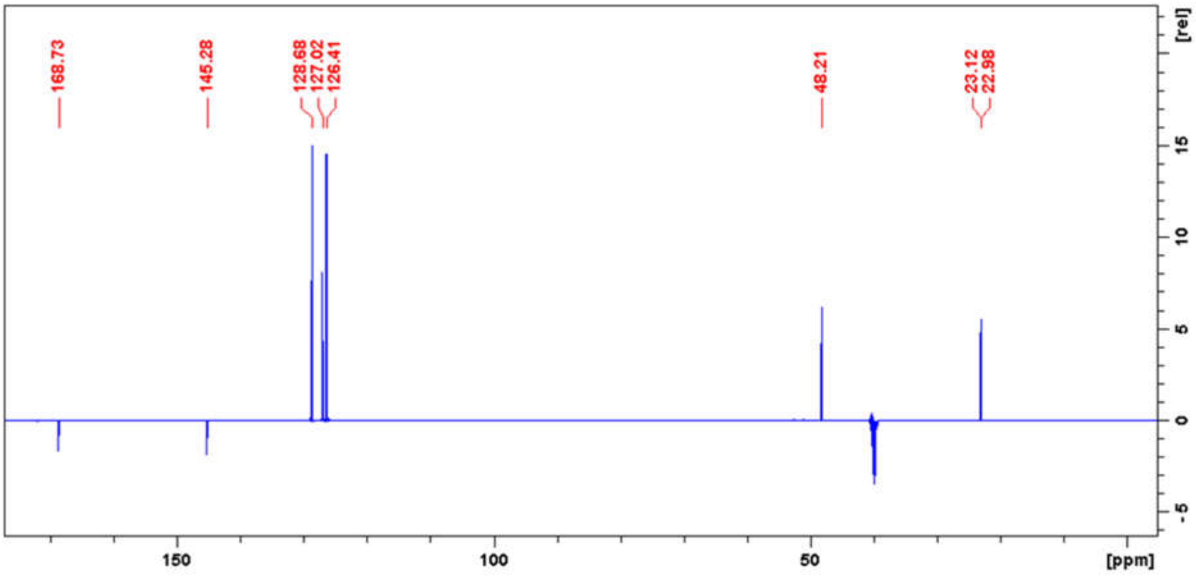

Figure S20. APT NMR spectrum of (R)-N-(1-phenylethyl)acetamide **26** measured in DMSO-d_6 at 298 K. $[\alpha]^{20}_{D}$ = + 149 (c=1.00, ethanol).

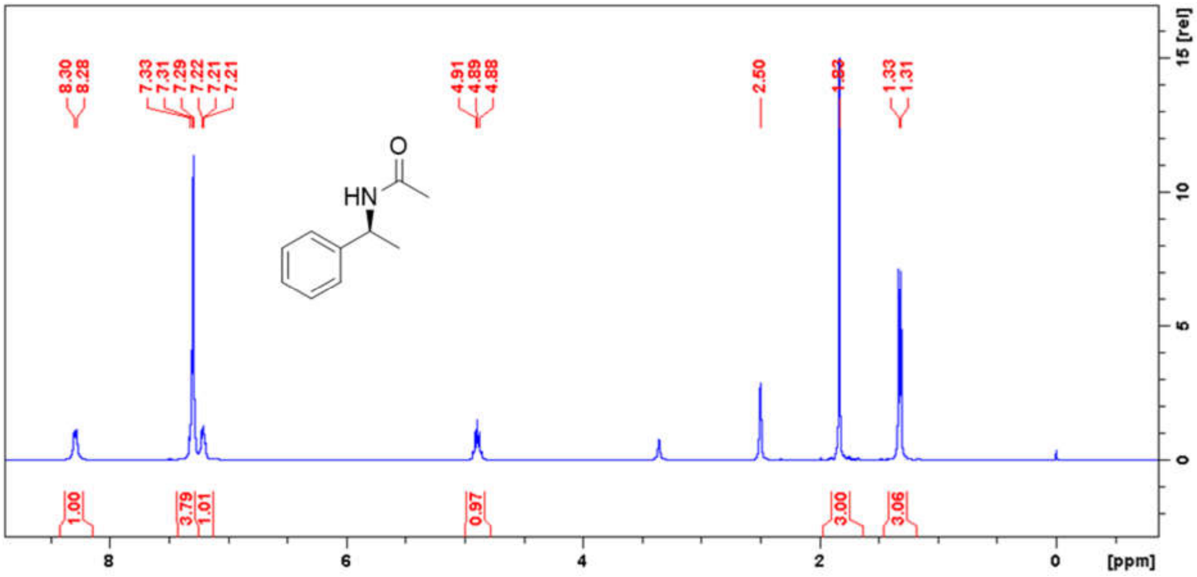

Figure S21. ^{1}H NMR spectrum of (S)-N-(1-phenylethyl)acetamide **28** measured in DMSO-*d*$_6$ at 298 K.

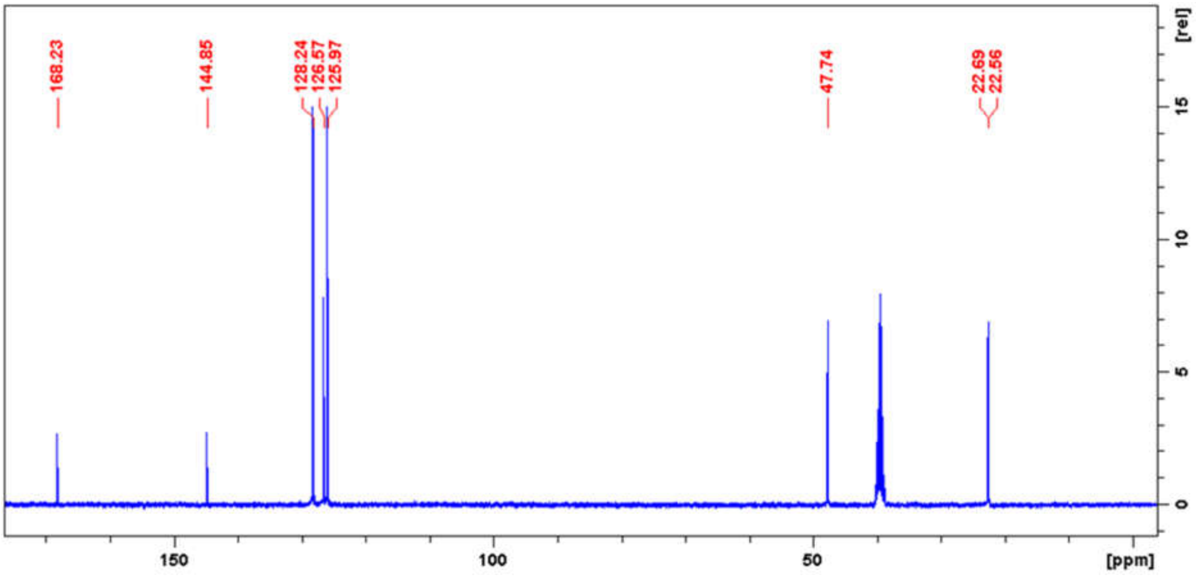

Figure S22. ^{13}C NMR spectrum of (S)-N-(1-phenylethyl)acetamide **28** measured in DMSO-*d*$_6$ at 298 K. $[\alpha]^{20}_{D}$ = - 150.1 (c=1.00, ethanol).

2. Image of the home made reactor

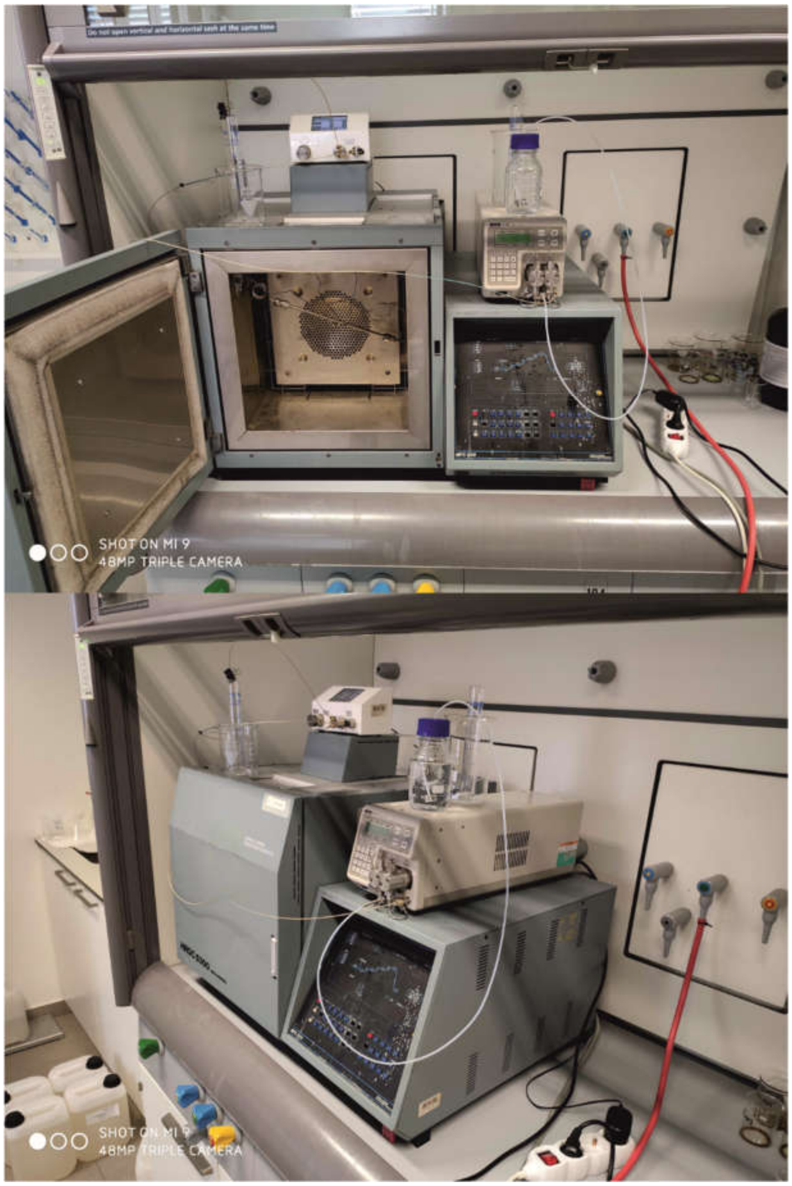

Figure S23. The reactor set-up used.
